# The Influence of Prior Obstetrical History on Current 17-Hydroxyprogesterone Caproate Use

**DOI:** 10.1155/2011/286483

**Published:** 2011-07-06

**Authors:** Carla E. Ransom, Jeanette R. Chin, Hilary A. Roeder, Tammy R. Sinclair, R. Phillips Heine, Amy P. Murtha

**Affiliations:** ^1^Department of Obstetrics and Gynecology, Duke University Medical Center (DUMC), P.O. Box 3967, Durham, NC 27710, USA; ^2^Department of Obstetrics and Gynecology, University of Utah, Salt Lake City, UT. 84132, USA

## Abstract

*Objective*. To determine if gestational age of prior preterm delivery influences a woman's receipt of 17-hydroxyprogesterone caproate (17-OHP-C). *Methods*. Retrospective cohort of women eligible for 17-OHP-C at Duke Obstetrics Clinic were identified by medical record review. Sociodemographic and clinical characteristics were abstracted. *Results*. Of 104 eligible subjects, 82 (78.8%) were offered 17-OHP-C. Of these, thirty-four (41.5%) declined. The median gestational age of the most recent preterm delivery was significantly lower among subjects who accepted 17-OHP-C as compared to those who declined (28.7 vs. 34.0 weeks, *P* = .02) and in subjects offered 17-OHP-C compared to those not offered 17-OHP-C (30.2 vs. 36.0 weeks, *P* = .03). Subjects not offered 17-OHP-C were more likely to have had an interval term delivery (31.8% vs. 9.7%, *P* = .009) *Conclusion*. Women with earlier preterm deliveries were more likely to be offered and accept 17-OHP-C. Prior obstetric history may influence both providers' and patients' willingness to discuss and/or accept 17-OHP-C.

## 1. Introduction

Preterm birth, defined as delivery prior to 37-week gestation, is the second leading cause of infant mortality in the United States after congenital malformations [1]. The incidence of preterm delivery continues to increase in this country such that it now exceeds 12% of all births [2]. The total national cost of care for premature babies is in excess of $13.6 billion annually [3]. Among survivors, the prevalence of both short, and long-term morbidities, including respiratory disease, neurodevelopmental problems, and gastrointestinal disease, is estimated to be as high as 60% [4].

The true cost of prematurity is only beginning to be understood. A recent study by Swamy et al. found diminished long-term survival and reproduction rates among individuals born prematurely in Norway between 1967 and 1988 [5]. It is becoming clear that, even if preterm infants surpass immediate obstacles, their overall long-term health is diminished. Despite extensive research in this field, the rate of preterm birth in the United States has increased over 20% in the past 20 years [2].

Recent randomized control trials have evaluated the role of 17-hydroxyprogesterone caproate (17-OHP-C) and progesterone gel or suppositories in the prevention of recurrent preterm birth [6–8]. Results from two trials suggest that administration of progestin to women at high-risk for preterm birth may decrease the recurrence risk by up to 35% [6, 7]. A more recent trial of progesterone vaginal gel versus placebo was not able to demonstrate a difference in the frequency of recurrent preterm birth ≤32 weeks in a high risk group of women [8]. The American College of Obstetricians and Gynecologists recommends that women at risk for recurrent preterm birth be considered candidates for progestin supplementation [9].

In a randomized trial of 17-OHP-C, Meis et al. found a preterm delivery rate with placebo of 55% much higher than the 37% that was originally predicted [7]. One proposed explanation for this finding was that the subjects enrolled in the trial might have been at particularly high risk for preterm delivery and not necessarily representative of the general population of women considered eligible for 17-OHP-C. Perhaps women with histories of earlier preterm deliveries would be more likely to enroll in a trial of progesterone. 

The primary objective in this study was to determine if gestational age of a prior preterm delivery influences *providers'* decisions to offer and *patients*' decisions to accept 17-hydroxyprogesterone caproate in the current gestation.

## 2. Materials and Methods

We conducted a retrospective cohort study of women eligible for 17-OHP-C at the Duke University Outpatient Obstetrics Clinic from January 2007 through June 2008. Approval was obtained from the Duke University Institutional Review Board. Subjects were identified by searching two independent obstetrics electronic clinical databases, and data were collected by chart abstraction.

All included subjects were pregnant and had a documented prior spontaneous, singleton preterm delivery due to either preterm labor or preterm premature rupture of membranes of less than 37 but more than 20-week gestation. Women with a multifetal gestation in the current pregnancy or with prior indicated preterm delivery were excluded from this analysis. Demographic and clinical characteristics collected include prior obstetric history, marital status, race, socioeconomic variables, pregnancy complications (preterm labor, gestational diabetes, abruption, antepartum bleeding, oligohydramnios, preeclampsia, gestational hypertension, or cerclage placement), pregnancy outcome, delivery route, chorioamnionitis, and indication for delivery. Neonatal outcomes collected included birth weight, sex, one- and five-minute APGAR scores, and congenital anomalies. If intermediate or intensive care was required, any further neonatal complications were recorded. The protocol at our institution is for the nurses to screen for 17-OHP-C eligibility and for the provider to enter into the electronic medical record if 17-OHP-C is offered. Patients with a prior history of indicated preterm delivery (such as for preeclampsia) were not eligible for 17-OHP-C at our institution and thus not included in this analysis. At our instituation, 17-OHP-C is started between 16/0 and 21/6 weeks and continues weekly until 34 weeks of gestation. We do not have a standard method for assessing compliance with progesterone therapy. Results were analyzed using *t*-test or Mann-Whitney for continuous variables and Fisher's exact test for categorical variables (Analyse-It, England, UK). For our primary objective, there were no missing data. Among the entire cohort, delivery data was unavailable for four patients (3.8%).

## 3. Results

Between January 2007 and June 2008, 104 subjects met eligibility criteria for 17-OHP-C. During the study period, approximately 1100 obstetrical patients were cared for in our clinical practice by twenty providers. Of the 104 eligible subjects, 82 (78.8%) were offered 17-OHP-C.  Table 1 describes the demographic and clinical characteristics of women eligible for 17-OHP-C. Those women offered 17-OHP-C were significantly younger and less educated than those women not offered 17-OHP-C, but otherwise the two groups did not differ in demographic or clinical characteristics. 

Among subjects eligible for 17-OHP-C, the median gestational age of the most recent preterm delivery was significantly lower for subjects offered 17-OHP-C as compared to those who were not offered 17-OHP-C (30.2 vs. 36.0 weeks, *P* = .03). In addition, subjects not offered 17-OHP-C were more likely to have had an interval term delivery, defined as a term delivery occurring after the index preterm delivery that qualified them for 17-OHP-C (31.8% vs. 9.7%, *P* = .009). 

Table 2 describes the characteristics of women who were offered 17-OHP-C. Of the 82 subjects offered 17-OHP-C, 48 (58.5%) accepted treatment. Subjects that accepted 17-OHP-C had a lower median gestational age in their most recent delivery as compared to those who declined (28.7 vs. 34.0 weeks, *P* = .02). The median gestational age of the earliest preterm delivery (that with the lowest gestational age) was also lower among subjects that accepted 17-OHP-C as compared to those who declined although this did not reach statistical significance (28.0 vs. 32.0 weeks, *P* = .11). In addition, subjects who declined 17-OHP-C tended to be more likely to have had an interval term delivery as compared to those who accepted (17.6% vs. 4.2%, *P* = .10). There was no significant difference in the median gestational age at delivery in the current gestation between subjects who received 17-OHP-C as compared to those who did not (37.0 vs. 37.2 weeks, *P* = .39).

## 4. Discussion

Our primary objective was to determine if gestational age at a prior preterm delivery impacts a provider decision to offer and/or a patient decision to accept 17-OHP-C. In this study, the median gestational age of the most recent preterm delivery was 6 weeks earlier in subjects offered 17-OHP-C as compared to those not offered 17-OHP-C and over 5 weeks earlier in subjects who accepted 17-OHP-C as compared to those who declined. These findings suggest that a woman pregnancy history seems to influence both providers and patients offered 17-OHP-C and whether the treatment is accepted. 

Despite the current enthusiasm for 17-hydroxyprogesterone caproate, few randomized trials have compared intramuscular progesterone or progestin to placebo for the prevention of preterm birth [7, 10–12]. In 1975, Johnson et al. provided some of the first evidence of an effect of 17-OHP-C on the prevention of recurrent preterm birth [11]. In 2003, Meis et al. found a reduction in the risk of preterm birth less than 37 weeks with the use of intramuscular 17-OHP-C among women at high risk for preterm delivery, but there was an unexpectedly high rate of preterm delivery in the place boarm [7]. This led to speculation that women enrolled in this trial were in some way at higher risk for recurrent preterm delivery than an average cohort of women with a history of preterm delivery. More complete demographics and pregnancy history of the patient group who refused randomization could help clarify the external validity of the Meis trial. 

A more recent randomized trial by O′Brien investigated vaginal progesterone for the prevention of preterm birth in a group of 659 women. This large study failed to find a difference between the placebo and study groups [8]. This was in contrast to an earlier trial by da Fonseca showing a decrease in preterm birth rate from 28.5% to 13.8% in the vaginal progesterone group versus placebo [6]. 

The purpose of our investigation was to examine the population of women potentially eligible for 17-OHP-C at our academic institution. In our clinical practice, there seems to be a subset of higher-risk women that are preferentially offered and more likely to accept 17-OHP-C. We believe our study population is representative of the population of women with prior preterm deliveries seen in many academic practices. As this population is predominantly single, African-American and on Medicaid, we believe this is a population of women at high risk for recurrent preterm birth. Compared to other studies, we had fairly broad inclusion criteria, which makes our study more generalizable to different populations. As with any retrospective study, our study is limited only to the patients captured via our medical record search. In addition, our study is not designed to evaluate the efficacy of 17-OHP-C. Other potential weaknesses include patient reporting bias on gestational age at prior preterm delivery. It is also possible that some patients were actually offered 17-OHP-C, but this was not documented. We did not assess for patient compliance with 17-OHP-C, which may be important in determining pregnancy outcomes. 

The limited rate of 17-OHP-C utilization in our institution could have many etiologies. These could include system errors in implementation of 17-OHP-C for appropriate patients. However, a reliable system already exists at our institution for indentifying potential candidates. Rather, the low utilization among this population more likely relates to either patient perception of the utility or risks of the drug and/or disagreement with the recommendations on drug implementation among providers. This study did not evaluate patient or provider motivations for drug utilization. 

Future research efforts are needed to determine the effect of patient and provider biases on the overall efficacy of 17-OHP-C. Studies could be directed at determining if there is a specific clinical phenotype that would benefit most from 17-OHP-C. Results in the literature are still mixed. In our study, 17-OHP-C was not commonly offered to women with a history of late preterm delivery. However, emerging evidence suggests that late preterm delivery causes greater morbidity than previously thought [13–15]. Although many providers do not offer 17-OHP-C to this group of women, it will be important not only to evaluate the effectiveness of 17-OHP-C in preventing recurrent preterm birth, but also to evaluate for a reduction of neonatal morbidity. Until these results are available, there will likely continue to be a bias among providers and patients with regards to offering and accepting progesterone supplementation.

##  Disclosure 

This work was presented at the annual meeting for the Society for Maternal Fetal Medicine, January 31, 2009, San Diego, Calif, USA.

## Figures and Tables

**Table 1:** Demographic and clinical characteristics of subjects eligible for 17- Hydroxyprogesterone caproate (17-OHP-C).

Patient characteristic	Offered 17-OHP-C (*n* = 82)	Not offered 17-OHP-C (*n* = 22)	*P* value
Maternal age (mean years ± SD)	**27.6 ± 5.2**	**31.7 ± 6.3**	**.002**
% African-American	59.7 (49/82)	72.7 (16/22)	.26
% Single	63.4 (52/82)	59.1 (13/22)	.71
% Medicaid insurance	73.2 (60/82)	54.5 (12/22)	.09
Years of school ≤12	**64.4 **(47/73)	**40.9 **(9/22)	**.04**
Preeclampsia or gestational hypertension	17.5 (14/80)	27.3 (6/22)	.31
Cerclage (all types)	12.3 (10/81)	9.1 (2/22)	1.00
Multiple (>1) prior preterm birth	39.0 (32/82)	27.3 (6/22)	.31
Etiology of index preterm birth due to preterm labor	58.0 (47/81)	59.1 (13/22)	.93
Interval term delivery since preterm birth	**9.7 **(8/82)	**31.8 **(7/22)	**.009**
Cesarean section (current pregnancy)	25.6 (20/78)	9.1 (2/22)	.14
Birth weight (mean gm ± SD)	2662.9 ± 744	2653.6 ± 923	.96
Gestational age of most recent preterm delivery*	**30.2 **(26.0–34.4)	**36.0 **(27.5–38.0)	**.03**
Gestational age of earliest preterm delivery*	29.2 (25.0–33.0)	31.5 (24.9–36.0)	.20
Gestational age at delivery of current gestation*	37.0 (35.2–39.1)	37.9 (36.5–39.0)	.87

*Data presented as median and IQR.

**Table 2:** Demographic and clinical characteristics of subjects offered 17-Hydroxyprogesterone caproate (17-OHP-C).

Patient characteristic	Received 17-OHP-C	Declined 17-OHP-C	*P* value
	(*n* = 48)	(*n* = 34)	
Maternal age (mean years ± SD)	27.7 ± 4.8	27.5 ± 5.8	.87
% African-American	54.2 (26/48)	67.6 (23/34)	.22
% Single	64.6 (31/48)	61.8 (21/34)	.79
% Medicaid Insurance	77.1 (37/48)	67.6 (23/34)	.34
% Years school ≤12	60.4 (26/43)	70.0 (21/30)	.40
% Preeclampsia or gestational hypertension (current pregnancy)	19.6 (9/46)	14.7 (5/34)	.76
% Cerclage (all types)	12.7 (6/47)	11.7 (4/34)	1.00
Multiple (>1) prior PTB	37.5 (18/48)	41.2 (14/34)	.74
Etiology of index preterm birth due to preterm labor	55.3 (26/47)	61.8 (21/34)	.56
Interval term delivery since preterm birth	4.2 (2/48)	17.6 (6/34)	.10
Cesarean section (current pregnancy)	28.3 (13/46)	21.9 (7/32)	.53
Birth weight (mean gm ± SD)	2571 ± 836	2595 ± 558	.47
Gestational age of most recent preterm delivery*	**28.7 **(25.3–32.8)	**34.0 **(28.3–36.0)	**.02 **
Gestational age of earliest preterm delivery*	28.0 (24.3–32.0)*	32.0 (25.2–34.0)*	.11
Gestational age at delivery of current gestation*	37.0 (35.2–38.4)*	37.2 (35.2–39.2)*	.39

*Data presented as median and IQR.
